# Current Response in Ca_*V*_1.3^–/–^ Mouse Vestibular and Cochlear Hair Cells

**DOI:** 10.3389/fnins.2021.749483

**Published:** 2021-12-08

**Authors:** Marco Manca, Piece Yen, Paolo Spaiardi, Giancarlo Russo, Roberta Giunta, Stuart L. Johnson, Walter Marcotti, Sergio Masetto

**Affiliations:** ^1^Department of Brain and Behavioral Sciences, University of Pavia, Pavia, Italy; ^2^School of Biosciences, University of Sheffield, Sheffield, United Kingdom; ^3^Sheffield Neuroscience Institute, University of Sheffield, Sheffield, United Kingdom

**Keywords:** auditory, vestibular, development, calcium current, potassium current, hair cells

## Abstract

Signal transmission by sensory auditory and vestibular hair cells relies upon Ca^2+^-dependent exocytosis of glutamate. The Ca^2+^ current in mammalian inner ear hair cells is predominantly carried through Ca_*V*_1.3 voltage-gated Ca^2+^ channels. Despite this, Ca_*V*_1.3 deficient mice (*Ca_*V*_1.3^–/–^*) are deaf but do not show any obvious vestibular phenotype. Here, we compared the Ca^2+^ current (*I*_*Ca*_) in auditory and vestibular hair cells from wild-type and *Ca_*V*_1.3^–/–^* mice, to assess whether differences in the size of the residual *I*_*Ca*_ could explain, at least in part, the two phenotypes. Using 5 mM extracellular Ca^2+^ and near-body temperature conditions, we investigated the cochlear primary sensory receptors inner hair cells (IHCs) and both type I and type II hair cells of the semicircular canals. We found that the residual *I*_*Ca*_ in both auditory and vestibular hair cells from *Ca_*V*_1.3^–/–^* mice was less than 20% (12–19%, depending on the hair cell type and age investigated) compared to controls, indicating a comparable expression of Ca_*V*_1.3 Ca^2+^ channels in both sensory organs. We also showed that, different from IHCs, type I and type II hair cells from *Ca_*V*_1.3^–/–^* mice were able to acquire the adult-like K^+^ current profile in their basolateral membrane. Intercellular K^+^ accumulation was still present in *Ca_*V*_1.3^–/–^* mice during *I*_*K,L*_ activation, suggesting that the K^+^-based, non-exocytotic, afferent transmission is still functional in these mice. This non-vesicular mechanism might contribute to the apparent normal vestibular functions in *Ca_*V*_1.3^–/–^* mice.

## Introduction

The inner ear houses the auditory and the balance organs. In mammals, the primary sensory cells are the inner hair cells (IHCs) of the cochlea, and the type I and type II hair cells of the vestibular system. Acoustic stimuli or head movements cause change in the hair cell membrane potential, which modulates Ca^2+^ inflow and related neurotransmitter (glutamate) exocytosis (Bonsacquet et al., 2006; Dulon et al., 2009; Songer and Eatock, 2013; Sadeghi et al., 2014; Vincent et al., 2014; Kirk et al., 2017). Both auditory and vestibular hair cells express voltage-gated L-type Ca^2+^ channels containing the pore-forming Ca_*V*_1.3 subunit (previously known as α1D), which are characterized by a negative voltage of activation (about −60 mV) and negligible voltage-dependent inactivation (Platzer et al., 2000; Bao et al., 2003; Johnson and Marcotti, 2008; Zampini et al., 2013). Pharmacologically, L-type (Ca_*V*_1.1 to Ca_*V*_1.4) Ca^2+^ channels are identified by their sensitivity to dihydropyridines (DHPs) such as nimodipine and nifedipine (antagonists) or BayK 8644 (agonist), which do not affect the other voltage-gated Ca^2+^ channels (Ca_*V*_2 and Ca_*V*_3). However, the Ca_*V*_1.3 subunit is relatively insensitive to DHP antagonists compared to Ca_*V*_1.1, 1.2, and 1.4 subunits (Koschak et al., 2001; Xu and Lipscombe, 2001).

The majority (∼90%) of the Ca^2+^ current in IHCs is carried by the Ca_*V*_1.3 subunit (Platzer et al., 2000; Jeng et al., 2020a). The nature of the remaining ∼10% of the Ca^2+^ current is still unknown (see Pangrsic et al., 2018), although previous work has indicated that it could be carried by the Ca_*V*_1.4 subunit (Brandt et al., 2003). Consistent with the critical role of Ca_*V*_1.3 channels in hair cell Ca^2+^ dependent exocytosis, *Ca_*V*_1.3^–/–^* mice are deaf (Platzer et al., 2000; Brandt et al., 2003), but do not show vestibular deficits (Platzer et al., 2000; Dou et al., 2004). A pharmacological study from rat semicircular canal crista hair cells has indicated that the level of expression of Ca_*V*_1.3 Ca^2+^ channels is comparable to that of the cochlear IHCs (Bao et al., 2003). However, vestibular utricle hair cells from *Ca_*V*_1.3^–/–^* mice appear to express a large residual Ca^2+^ current (∼50%, Dou et al., 2004), which could potentially drive some signal transmission to the afferent fibers, at least during linear horizontal head accelerations. Currently, it is unknown whether a substantial residual Ca^2+^ current is also expressed in *Ca_*V*_1.3^–/–^* semicircular canal hair cells, which could compensate for rotation-related reflexes (e.g., vestibulo-ocular reflexes), and whether its size is similar between type I and type II hair cells. The possible presence of large residual Ca^2+^ currents in vestibular hair cells from *Ca_*V*_1.3^–/–^* mice, together with the recently identified non-quantal, Ca^2+^-independent, signal transmission at type I hair cell synapses (Eatock, 2018) could contribute, at least in part, to the milder vestibular phenotype present in *Ca_*V*_1.3^–/–^* mice. Non-quantal transmission, which has not been reported in auditory hair cells, involves intercellular K^+^ accumulation in the synaptic cleft occurring during the activation of a hyperpolarizing-activated outward rectifying K^+^ current *I*_*K,L*_ (Lim et al., 2011; Contini et al., 2012, 2017, 2020; Spaiardi et al., 2017, 2020a).

Here, we have performed whole-cell patch clamp recordings from IHCs and both type I and type II hair cells from the mammalian crista of *Ca_*V*_1.3^–/–^* mice using the same experimental conditions in terms of extracellular Ca^2+^ and temperature. We have found that both IHCs and vestibular hair cells from *Ca_*V*_1.3^–/–^* mice have < 20% of residual Ca^2+^ current compared to control mice. Thus, differences in Ca_*V*_1.3 Ca^2+^ channel expression among vestibular organs (saccule vs. semicircular canals) may exist, possibly allowing hair cells from some vestibular organs to retain some Ca^2+^-dependent neurotransmitter exocytosis. We have also found that, different from cochlear IHCs, Ca_*V*_1.3 Ca^2+^ channels are not required for the normal maturation of the biophysical properties (K^+^ channel expression) of both type I and type II hair cells. Since *I*_*K,L*_ and intercellular K^+^ accumulation in the synaptic cleft of type I hair cells were normal in *Ca_*V*_1.3^–/–^* mice, non-quantal signal transmission might also contribute to the vestibular functioning of *Ca_*V*_1.3^–/–^* mice.

## Materials and Methods

### Ethics Statement

All animal work was performed at the University of Sheffield (United Kingdom), licensed by the Home Office under the Animals (Scientific Procedures) Act 1986 (PPL_PCC8E5E93) and approved by the University of Sheffield Ethical Review Committee (180626_Mar). For all *in vitro* work, mice were culled by cervical dislocation, which is a schedule 1 method.

### Tissue Preparation

Experiments were performed using *Ca_*V*_1.3* knockout mice (*Ca_*V*_1.3^–/–^*) on a C57BL/6N background and control mice (littermate heterozygous or C57BL/6N). Mice from both sexes were used and ranging from postnatal day 4 (P4) to P35. The semicircular canals with their *ampullae* and cochleae (apical turn) were dissected out from the inner ear as reported previously (Jeng et al., 2020a,^2021^; Spaiardi et al., 2020a; Carlton et al., 2021), using an extracellular solution composed of (in mM) 135 NaCl, 5.8 KCl, 1.3 CaCl_2_, 0.9 MgCl_2_, 0.7 NaH_2_PO_4_, 5.6 D-glucose, and 10 HEPES-NaOH. Sodium pyruvate (2 mM), amino acids, and vitamins were added from concentrates (Thermo Fisher Scientific, United Kingdom). The pH was adjusted to 7.5 (osmolality ∼308 mmol kg^–1^). The dissected organs were fixed at the bottom of the recording chamber by a nylon-meshed silver ring and were continuously perfused with the above extracellular solution (0.5 ml/min) using a peristaltic pump (Masterflex L/S, Cole Palmer, United States). Hair cells were viewed using a upright microscopes (Olympus BX51; Leica DM-LFS) equipped with Nomarski Differential Interface Contrast (DIC) optics with a 60X or 64X water immersion objective and x15 eyepieces.

### Whole-Cell Electrophysiology

Voltage-clamp whole-cell experiments were performed at room temperature (18–22°C) for K^+^ current recordings, and near-body temperature (32–36°C) for Ca^2+^ currents recordings using an Optopatch amplifier (Cairn Research Ltd., United Kingdom) as previously described (Johnson and Marcotti, 2008; Jeng et al., 2020b; Spaiardi et al., 2020b). The patch pipettes were pulled to 2–3 MΩ tip resistance from soda glass capillaries (Hilgenberg, Germany) and coated with surf-wax (Mr. Zoggs SexWax, United States) to minimize the fast capacitance transient across the wall of the patch pipette. For K^+^ current recordings, the patch pipette filling solution contained (in mM): KCl 131, Na_2_-Phosphocreatine 10, MgCl_2_ 3, EGTA-KOH 1, Na_2_ATP 5, and HEPES 5; pH adjusted to 7.28 with KOH (osmolality was 294 mmol kg^–1^). For Ca^2+^ current recordings, the pipette intracellular solution contained (in mM): 106 Cs-glutamate, 20 CsCl, 3 MgCl_2_, 1 EGTA-CsOH, 5 Na_2_ATP, 0.3 Na_2_GTP, 5 HEPES-CsOH, 10 Na_2_-phosphocreatine, pH 7.3 with CsOH (294 mmol kg^–1^). Data acquisition was controlled by pClamp software using a Digidata board (Molecular Devices, United States). Recordings were low-pass filtered at either 2.5 or 5 kHz (8-pole Bessel), sampled at 5, 10, or 100 kHz and stored on computer for off-line analysis using Clampfit (Molecular Devices, United States) and Origin (OriginLab, United States) software. Membrane potentials reported in the text and figures were corrected for the uncompensated residual series resistance (*R*_*s*_) and the liquid junction potential (LJP), which was either −4 mV for the K^+^-based and −11 mV for the Cs^+^-glutamate-based intracellular solution, measured between electrode and bath solutions. For K^+^ current recordings from vestibular hair cells, *R*_*s*_ was calculated off-line from the capacitive artifact elicited by applying a voltage step from either −74 to −64 mV in type II and −124 to −44 mV in type I hair cells. The different voltage step was used in order to minimize artifact contamination by inward and outward rectifier voltage-gated channels in the two hair cell types (Spaiardi et al., 2017). Voltage clamp protocols are referred to a holding potential of −64 mV for the K^+^-based intracellular solution or −91 mV for the Cs^+^-based intracellular solution.

For Ca^2+^ current recordings, the composition of the extracellular solution contained (in mM): NaCl 101, CaCl_2_ 5, CsCl 5.8, MgCl_2_ 0.9, HEPES 10, glucose 5.6, tetraethylammonium (TEA) 30, 4-aminopyridine (4AP) 15 (pH adjusted with HCl was 7.5; osmolality: 312 mOsm/kg). The higher Ca^2+^ concentration (5 mM) was used to better visualize the Ca^2+^ current in *Ca_*V*_1.3^–/–^* mice. Extracellular TEA, 4AP, and intracellular Cs^+^ (see above) were used to block the K^+^ channels (cochlear IHCs: Marcotti et al., 2003; Jeng et al., 2020b,^2021^; vestibular hair cells: Rennie and Correia, 1994; Biel et al., 2009). Moreover, the K^+^ channel blockers linopirdine (80 μM; Tocris, United Kingdom) was also used to block *I*_*K,n*_ in adult IHCs (Marcotti et al., 2003). In some experiments, CdCl_2_ 0.1 mM was also added to the above extracellular solution to block the Ca^2+^ current (Hille, 2001) in vestibular hair cells. The amplitude of the Ca^2+^ current was measured by either subtracting the linear leakage current component, measured between −81 and −91 mV, or the current blocked by Cd^2+^ from the current recorded in the presence of TEA and 4AP (see above).

### Statistics Analysis

Statistical comparisons of means were made by Student’s two-tailed *t*-test or, for multiple comparisons, analysis of variance (one-way or two-way ANOVA) was applied. *P* < 0.05 was selected as the criterion for statistical significance. Mean values are quoted as means ± SD.

## Results

### Potassium Currents Recorded From Type I and Type II Crista Hair Cells From Control and *Ca_*V*_1.3^–/–^* Mice

**FIGURE 1:**
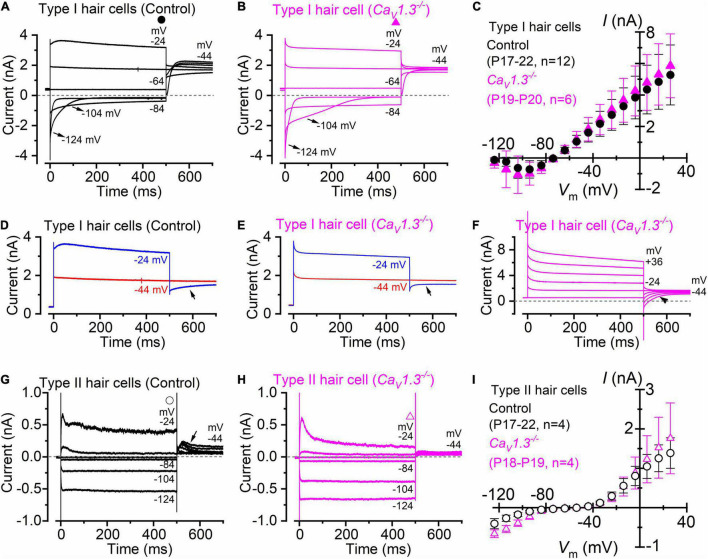
Type I and type II crista hair cells express normal K^+^ currents in control and *Ca_*V*_1.3*^–/–^ adult mice. **(A,B)** Membrane current responses from type I hair cells of control [**(A)**, P18] or *Ca_*V*_1.3*^–/–^ [**(B)** P20] adult mice were performed by applying 500 ms depolarizing voltage steps of 10 mV nominal increment from −124 mV, starting from a holding potential of −64 mV. For clarity, only a few test potentials are shown. **(C)**, Steady-state current-voltage (*I-V*) curves obtained from type I hair cells of control (P17–22) and *Ca_*V*_1.3*^–/–^ (P19–20) mice. **(D,E)** Current responses from panels **(A,B)** but at only two membrane potentials that better emphasize the tail currents. **(F)** Potassium current recorded from a type I hair cell of a *Ca_*V*_1.3^–/–^* mouse (P19). The tail currents at −44 mV were elicited following 500 ms voltage steps between −64 to 40 mV in 20 mV nominal increments (actual membrane potential is shown near some of the traces). The instantaneous tail current reverse (arrow) following the two most depolarized voltage steps. The horizontal dashed line represents the zero-current level. For a detailed explanation of the above phenomenon, see Spaiardi et al. (2017). **(G,H)** Membrane current responses from type II hair cells of control [**(G)**, P17] or *Ca_*V*_1.3*^–/–^ [**(H),** P19] adult mice obtained using the voltage protocol described above. Note that the larger tail outward currents at −44 mV [arrow in panel **(G)]**, elicited following the 500 ms hyperpolarizing voltage steps, were due to progressive removal of *I*_*K,A*_ inactivation. **(I)** Steady-state *I-V* curves obtained from type I hair cells of control (P17–22) and *Ca_*V*_1.3*^–/–^ (P18–19) mice. In panels **(C,I)** values are shown as mean ± S.D.

Type I hair cells of the *ampullae* sensory epithelium (the *crista*) exhibit a large low-voltage activated outward rectifying K^+^ currents, named *I*_*K,L*_ (Rennie and Correia, 1994; Rüsch and Eatock, 1996), which was recorded in both adult wild-type (Figure 1A) and *Ca_*V*_1.3^–/–^* mice (Figure 1B). Since *I*_*K,L*_ is almost completely activated at −60 mV, hyperpolarizing voltages from the holding potential of −64 mV produced deactivating tail currents (e.g., −104 and −124 mV: Figures 1A,B), while depolarizations elicit an instantaneous increase of the outward currents. The mean steady-state current-voltage (*I*-*V*) relationship for the total current recorded in type I hair cells was not significantly different between wild-type (P17-P22; *n* = 12) and *Ca_*V*_1.3^–/–^* mice (P19-P20; *n* = 6) (*P* = 0.9742, *F* = 0.4149, DFn = 15, two-way ANOVA, Figure 1C). The negligible steady-state current at −124 mV is consistent with *I*_*K,L*_ being fully deactivated at this potential, and with the absence of inward rectifier currents in mouse *crista* type I hair cells (Spaiardi et al., 2020a). Note that the outward tail currents in type I hair cells, which were recorded at −44 mV, were smaller following the voltage step to −24 mV than those at −44 mV in both wild-type (Figures 1A,D) and *Ca_*V*_1.3^–/–^* mice (Figures 1B,E,F). The presence of progressively smaller outward tail current following larger outward K^+^ currents (Figures 1D,E), which in some cases includes tail current reversal (Figure 1F), is consistent with intercellular (e.g., in the calyceal synaptic cleft) K^+^ accumulation inducing a shift in the K^+^ equilibrium potential (Lim et al., 2011; Contini et al., 2012, 2017, 2020; Spaiardi et al., 2020a). Progressive intercellular K^+^ accumulation during depolarization is also responsible for the “apparent” inactivation of the outward current, which is due to the progressive decrease of the driving force for K^+^ to exit the hair cells (e.g., Spaiardi et al., 2017).

Different from the type I hair cells, the macroscopic current recorded from adult type II hair cells is not dominated by *I*_*K,L*_ (Figures 1G,H), but instead express several other currents that have been previously described in great details (Meredith and Rennie, 2016). This include a transient (*I*_*K,A*_) and a delayed rectifying (*I*_*K,v*_) outward K^+^ current, an inward rectifying K^+^ current (*I*_*K,1*_), and the mixed inward rectifying Na^+^/K^+^ current (*I*_*h*_). All these currents were evident from the inward and outward current profile recorded from type II hair cells of *Ca_*V*_1.3^–/–^* mice (Figure 1H). The mean steady-state *I*-*V* for the total current recorded in type II hair cells was also not significantly different between wild-type (P17-P22; *n* = 4) and *Ca_*V*_1.3^–/–^* mice (P18-P19; *n* = 4) (*P* = 0.7010, *F* = 0.7756, DFn = 15, two-way ANOVA, Figure 1I).

The above results demonstrate that the absence of the *Ca_*V*_1.3* Ca^2+^ channel subunit does not impair the normal developmental acquisition of voltage-dependent K^+^ currents in vestibular type I and II hair cells in the *crista*. This is different from the cochlea, where the normal expression of the K^+^ currents characteristic of adult inner hair cells (IHCs), which are the fast activating BK current *I*_*K,f*_ and negatively activating delayed rectifier *I*_*K,n*_ (Kros et al., 1998; Marcotti et al., 2003; Oliver et al., 2003), was either prevented (apical-coil) or reduced (basal-coil) in *Ca_*V*_1.3^–/–^* mice (Brandt et al., 2003; Jeng et al., 2020a).

### Calcium Currents in Type I and Type II Hair Cells in Control and *Ca_*V*_1.3^–/^*^–^ Mice

**FIGURE 2:**
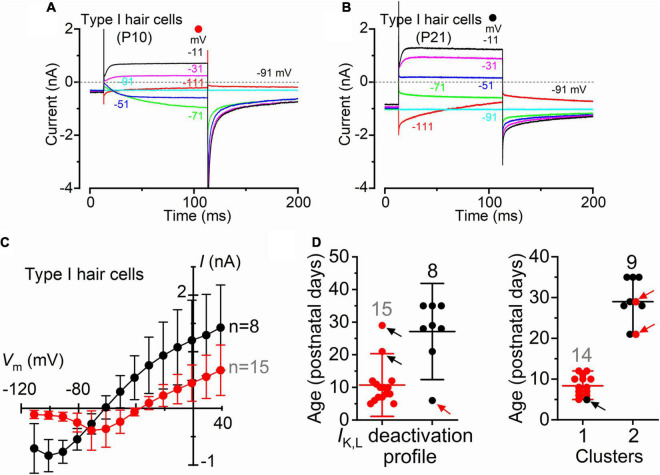
Type I crista hair cells can be identified by their large permeability to Cs^+^ in wild-type neonatal and adult mice. **(A,B)** Representative membrane current responses recorded from type I hair cells of control mice of different age with Cs^+^ as main ion carrier in the intracellular solution [**(A),** P10; **(B),** P21]. Currents were elicited by 500 ms depolarizing voltage steps of 10 mV nominal increment from −111 mV starting from a holding potential of −91 mV. For clarity, only a few test potentials are shown. **(C)** Steady-state *I*-*V* curves obtained from type I hair cells arbitrarily subdivided into two groups depending if the inward current through the K,L channels was (red), or was not (black), completely deactivated at −111 mV. Values are shown as mean ± SD. **(D)** Left: Age distribution of type I hair cells expressing *I*_*K,L*_ that was (red), or was not (black), completely deactivated at −111 mV. Each symbol refers to the steady-state value of *I*_*K,L*_ measured in each cell at −111 mV. Right: same data as in the left panel but subdivided into type I hair cells that are completely deactivated (red) and not deactivated (black) at −111 mV clusters using k-means clustering. Arrows indicate the hair cells that are located in one cluster but have *I*_*K,L*_ properties that fit best the other cluster.

Although type I and type II hair cells are morphologically distinct, this is not always visible when working with the intact organ. Therefore, the correct identification of type I hair cells relies on the presence of *I*_*K,L*_ (Figure 1). Recordings were performed using intracellular Cs^+^, which is known to block *I*_*h*_ (Biel et al., 2009) and the outward rectifying K^+^ currents (Bao et al., 2003) in type II hair cells while having little effect on *I*_*K,L*_ (Griguer et al., 1993; Rüsch and Eatock, 1996; Chen and Eatock, 2000; Rennie and Correia, 2000). An additional consideration affecting the correct identification of *I*_*K,L*_ is its state of deactivation at the holding potential used for the recordings, which is generally set at −90 mV. Figures 2A,C shows representative current responses recorded from a type I hair cell with a largely deactivated *I*_*K,L*_, as indicated by the small inward current at −91 mV, and the small instantaneous current upon depolarizing and hyperpolarizing voltage steps. In the example shown in Figures 2B,C, however, *I*_*K,L*_ is largely activated at −91 mV and exhibits large instantaneous currents. Although this variability has previously been reported (Hurley et al., 2006; Spaiardi et al., 2017), we found that the reversal potential for the macroscopic current was significantly more hyperpolarised in type I hair cells showing a largely activated *I*_*K,L*_ at −91 mV (Figure 2C). While a deactivated *I*_*K,L*_ at the holding potential was primarily recorded in early postnatal hair cells (*n* = 15), more mature cells tend to exhibit a largely activated current (*n* = 8) (Figure 2D). Since the reversal potential of the macroscopic K^+^ current in type I hair cells is primarily determined by the ions flowing through the K,L channels, it should reach values near the reversal potential of the mixed Cs^+^/K^+^ current (called a “biionic potential”; see Hille, 2001) through K,L channels in the presence of a large *I*_*K,L*_ and intracellular Cs^+^ (Spaiardi et al., 2020a). Given the reported permeability ratio of Cs^+^ to K^+^ of about 0.31 (Rüsch and Eatock, 1996; Spaiardi et al., 2020a), the reversal potential should be close to −40 mV. The finding that the reversal potential in older hair cells (Figure 2C) is close to −60 mV could indicate that the relative permeability of K,L channels to Cs^+^ is likely to be larger than that previously estimated, at least in more mature type I hair cells. However, the presence of a residual calyx might also produce a shift of the mixed Cs^+^/K^+^ equilibrium potential toward more negative voltages during inward currents (Spaiardi et al., 2017, 2020a). This would imply a tighter attachment of the calyx to more mature type I hair cells.

The above results are consistent with *I*_*K,L*_ increasing in amplitude and activating at more hyperpolarized potentials during post-natal development, as also previously shown in rat type I hair cells (Hurley et al., 2006). Different K^+^ channels subunits have also been found to be expressed by rodent type I hair cells during postnatal development which may account for the above changes (Hurley et al., 2006; Spitzmaul et al., 2013).

**FIGURE 3:**
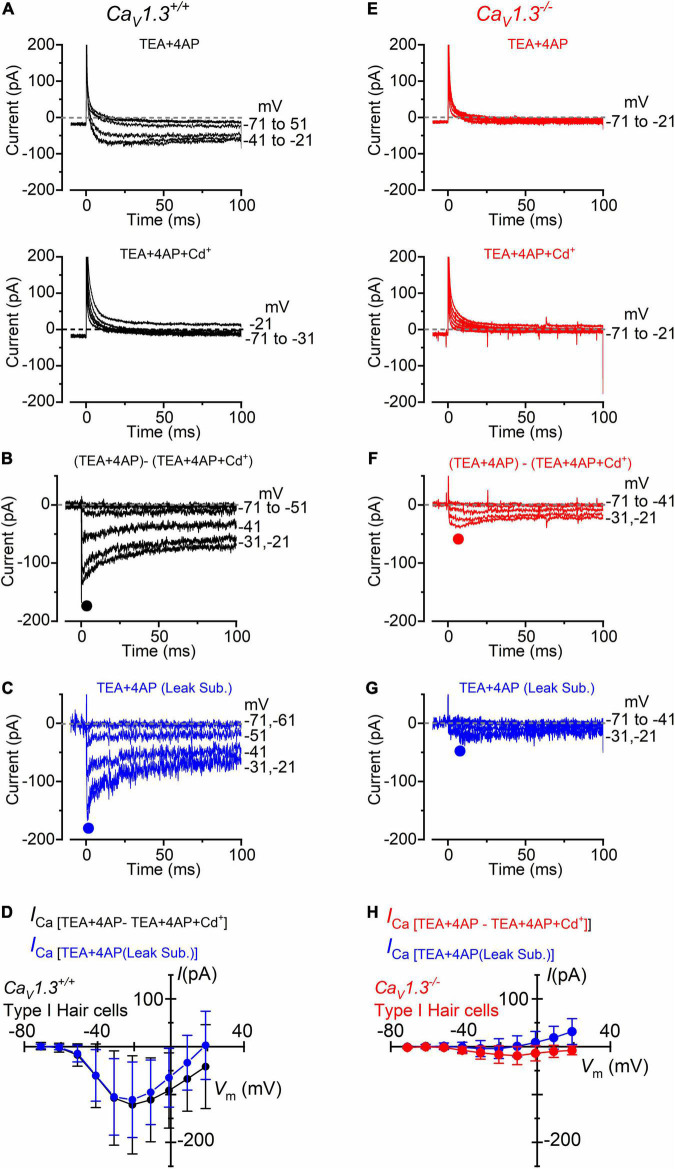
Calcium currents in type I crista hair cells from wild-type and *Ca_*V*_1.3*^–/–^ mice. **(A)** Upper panel: current recorded from a type I hair cell of a wild-type mouse (P5) in the presence of 30 mM TEA and 15 mM 4AP in the extracellular solution and Cs^+^ as the main ion carrier in the intracellular solution. Currents were elicited by 100 ms depolarizing voltage steps of 10 mV nominal increment (holding potential of −91 mV). For clarity, only a few test potentials are shown. Lower panel: current recorded from the same cell and solution shown in the top panel but with the additional 0.1 mM Cd^2+^ (Ca^2+^ channel blocker). **(B)** Inward Ca^2+^ current from the same wild-type hair cell shown in panel **(A)** obtained by subtracting the current recorded in extracellular TEA, 4AP, and 0.1 mM Cd^2+^ to that recorded in TEA and 4AP. **(C)** Inward current obtained by leakage subtraction of the current recorded from the cell in panel **(A)**, upper panel. **(D)** Peak inward *I*-*V* curves for the current obtained as the Cd^2+^-sensitive current (black symbols, *n* = 8) or after leakage subtraction (blue symbols; *n* = 17). **(E–G)** Currents recorded from a type I hair cell from a *Ca_*V*_1.3*^–/–^ (P5) mouse obtained as described in panels **(A–C)**. **(H)** Peak inward *I*-*V* curves for the current obtained as the Cd^2+^-sensitive current (red symbols, *n* = 10) or after leakage subtraction (blue symbols; *n* = 36). Values in panels **(D,H)** are shown as mean ± SD.

Following the positive identification of the patched hair cell as type I, a small inward current (Figure 3A, upper panel) became visible when superfusing the cells with an extracellular solution containing the K^+^ channel blockers TEA and 4AP, in the presence of 5 mM Ca^2+^ (see section “Materials and Methods”). In a subset of type I hair cells (*n* = 8), we found that the addition of 0.1 mM Cd^2+^ to the extracellular solution fully blocked the inward current, confirming its identity as *I*_*Ca*_ (Figure 3A, lower panel). The isolated *I*_*Ca*_ was obtained by subtracting the current recorded in the presence of TEA, 4AP, and Cd^2+^ to that obtained in the absence of Cd^2+^ (Figure 3B). Under this experimental condition, the inward current activated at about −61 mV and peaked near −21 mV (Figures 3B,D). Comparable results were obtained when *I*_*Ca*_ was isolated by performing the leakage-subtraction procedure (see section “Materials and Methods”) to the currents recorded in TEA and 4AP (Figures 3C,D).

In *Ca_*V*_1.3^–/–^* mice, the inward current was barely visible in type I hair cells when the extracellular solution contained TEA, 4AP, and 5 mM Ca^2+^ (Figure 3E). Following the isolation of *I*_*Ca*_ with Cd^2+^ (Figure 3F), or after leakage subtraction (Figure 3G), it became more easily detectable. The inward current peaked near −11 mV (Figure 3H). In type I hair cells, the mean peak *I*_*Ca*_ after Cd^2+^-subtraction was 121 pA in wild-type and 19 pA in *Ca_*V*_1.3^–/–^* mice (15.7% of the wilt-type value).

**FIGURE 4:**
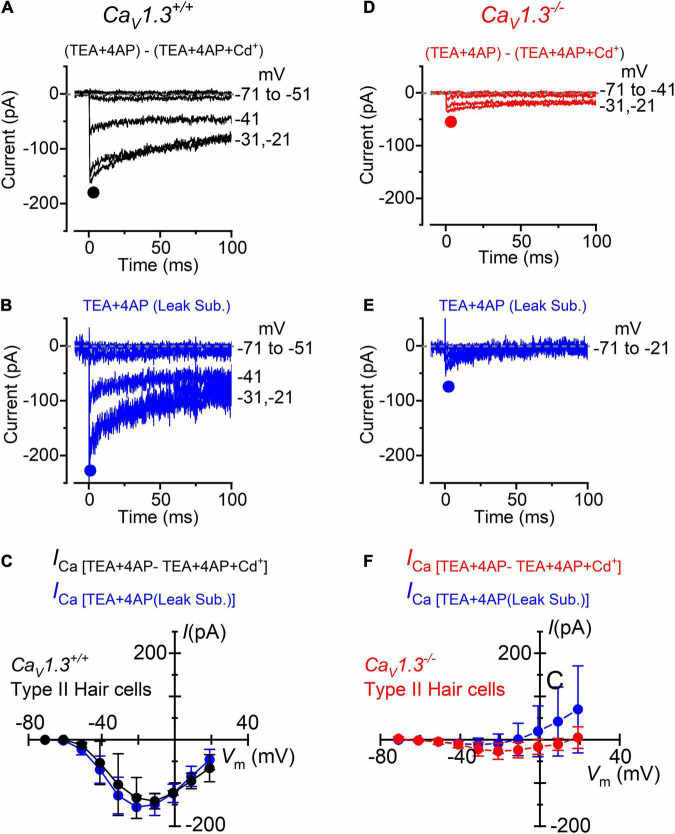
Calcium currents in type II crista hair cells from wild-type and *Ca_*V*_1.3*^–/–^ mice. **(A,B)** Inward current recorded from a type II hair cell of a wild-type mouse (P5) using the same experimental conditions described in Figures 3B,C. For clarity, only a few test potentials are shown. **(C)** Peak inward *I*-*V* curves for the Cd^2+^-sensitive current (black symbols, *n* = 2) or for the current after leakage subtraction (blue symbols; *n* = 5). **(D,E)** Inward currents recorded from a type II hair cell from a *Ca_*V*_1.3*^–/–^ (P5) mouse obtained as described in panels **(A,B)**. **(F)** Peak inward *I*-*V* curves for the current obtained as the Cd^2+^-sensitive current (red symbols, *n* = 3) or after leakage subtraction (blue symbols; *n* = 12). Values in panels **(C,F)** are shown as mean ± SD.

For type II hair cells, the isolation of the Ca^2+^ current was performed as shown in Figure 3, using either the isolated Cd^2+^-procedure (Figures 4A,D) or after leakage subtraction (Figures 4B,E) in both wild-type and *Ca_*V*_1.3^–/–^* mice, respectively. In WT type II hair cells the amplitude of the Cd^2+^-sensitive current (*I*_*Ca*_: Figures 4A,C) was not significantly different to that obtained by leakage-subtraction (Figures 4B,C: *P* < 0.9336, *F* = 0.3916, DFn = 9, two-way ANOVA), or to the Cd^2+^-sensitive current measured in wild-type type I hair cells (Figures 3B,D: *P* < 0.8457, two-way ANOVA). The size of the Cd^2+^-sensitive *I*_*Ca*_ recorded in type II hair cells of *Ca_*V*_1.3^–/–^* mice (Figures 4D,F) was very similar to that measured in type I hair cells (Figures 3F,H, *P* < 0.7677, *F* = 0.6319, DFn = 9, two-way ANOVA). In type II hair cells, the mean peak *I*_*Ca*_ after Cd^2+^-subtraction was 142 pA in wild-type and 27 pA in *Ca_*V*_1.3^–/–^* mice (19.0% of the wild-type value).

**FIGURE 5:**
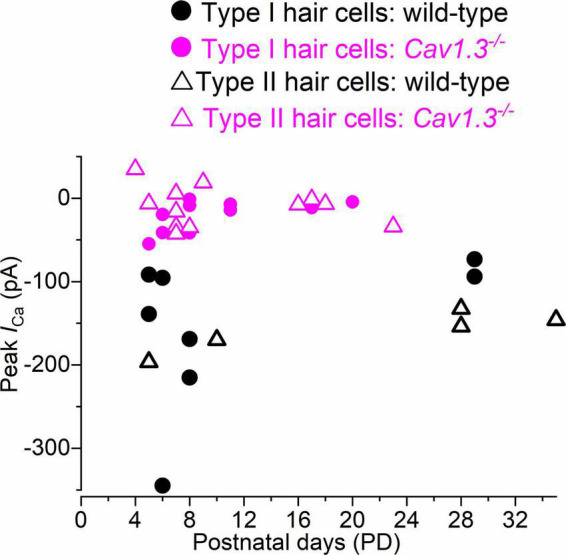
Calcium current in type I and type II hair cells as a function of postnatal age in mice. Peak of the Ca^2+^ current measured as a function of postnatal age in type I and type II hair cells from both wild-type and *Ca_*V*_1.3*^–/–^ mice. Single data points are shown.

We tested whether the size of *I*_*Ca*_ changed with age in hair cells from wild-type and *Ca_*V*_1.3^–/^*^–^ mice. The peak *I*_*Ca*_ amplitude, which was detected between −21 and −10 mV, was plotted as a function of postnatal day (Figure 5). The size of *I*_*Ca*_ in both type I and type II hair cells appears to be comparable between early and late postnatal ages in both wild-type and *Ca_*V*_1.3^–/–^* mice.

### Calcium Currents in the Inner Hair Cells of the Mouse Cochlea From *Ca_*V*_1.3^–/–^* Mice

**FIGURE 6:**
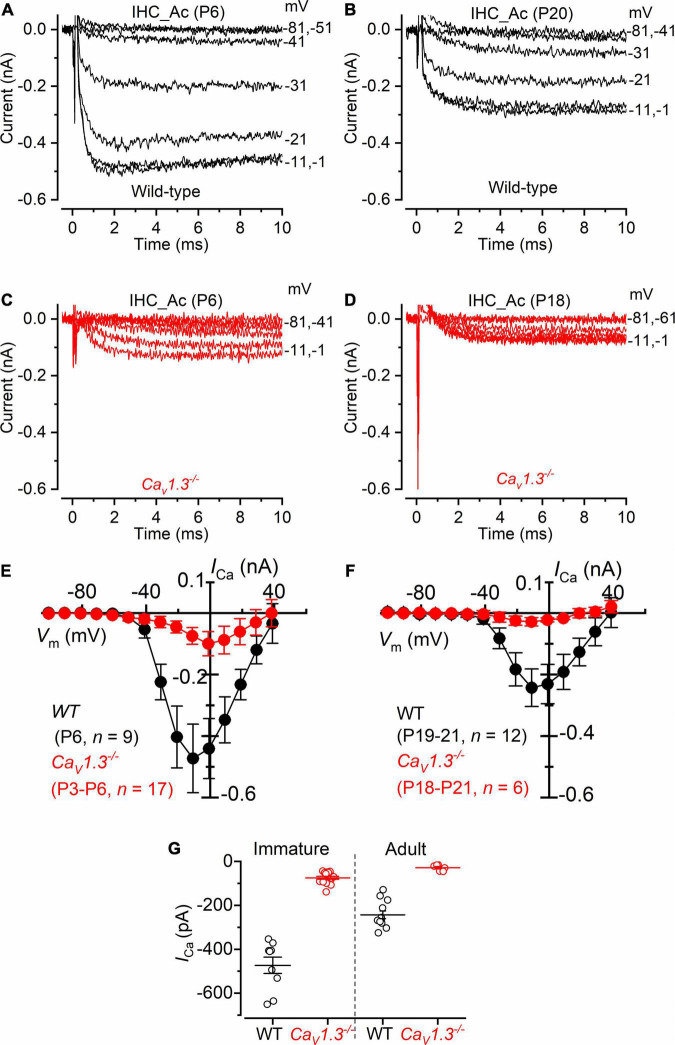
Calcium currents in IHCs from *Ca_*V*_1.3^–/–^* mice. **(A,B)** Calcium currents recorded from immature [**(A)**, P6] and adult [**(B)**, P20] IHCs of wild-type mice. Currents were elicited by depolarizing voltage steps of 10 mV increments (10 ms in duration) starting from the holding potential of −81 mV. **(C,D)** Calcium currents from immature [**(C),** P6] and adult [**(D)**, P18] IHCs of *Ca_*V*_1.3^–/–^* mice. For clarity, in panels **(A–D)** only some of the traces are shown. Actual test potentials, corrected for voltage drop across uncompensated *R*_*s*_, are shown next to the traces. **(E,F),** *I*-*V* curves for the inward Ca^2+^ current in immature **(E)** and adult **(F)** IHCs from both wild-type and *Ca_*V*_1.3^–/–^* mice. **(G)** Peak Ca^2+^ current in both genotypes at immature and adult IHCs. One-way ANOVA (overall: *P <* 0.0001, *F* = 98.98, DFn = 43) Tukey’s post-test analysis: wild-type immature (−473 ± 112 pA, *n* = 9) vs. *Ca_*V*_1.3^–/–^* immature (−74 ± 27 pA, *n* = 17), *P <* 0.0001; wild-type adult (−243 ± 61 pA, *n* = 12) vs. *Ca_*V*_1.3^–/–^* adult (−28 ± 14 pA, *n* = 6), *P <* 0.001; wild-type immature vs. wild-type adult, *P <* 0.001; wild-type immature vs. *Ca_*V*_1.3^–/–^* adult, *P <* 0.001; *Ca_*V*_1.3^–/–^* immature vs. wild-type adult, *P <* 0.001; *Ca_*V*_1.3^–/–^* immature vs. *Ca_*V*_1.3^–/–^* adult, *P >* 0.05. Data are reported as mean ± SD.

We investigated *I*_*Ca*_ in cochlear IHCs by performing whole-cells recordings at 32–36 °C and with the same solutions used for the above vestibular hair cell recordings (intracellular Cs^+^, extracellular TEA and 4AP, and 5 mM Ca^2+^ (Figure 6). About 10% of the total *I*_*Ca*_ in apical IHCs has been shown to be carried by Ca^2+^ channels other than Ca_*V*_1.3 (Platzer et al., 2000; Michna et al., 2003). Indeed, we found that the size of *I*_*Ca*_, measured after leakage-subtraction, was largely reduced in IHCs from *Ca_*V*_1.3^–/–^* mice compared to littermate controls at both immature (*P* < 0.0001, *F* = 63.70, DFn = 9) and adult ages (Figure 6G, *P* < 0.0001, *F* = 20.33, DFn = 9, between the same voltage range used for the vestibular hair cells: −61 to +29 mV, two-way ANOVA). The nature of the residual Ca^2^ channels in immature (∼16% of the wild-type value) and adult (∼12%) IHCs is unknown, but previous suggestions included Ca_*V*_1.4 (Brandt et al., 2003) or, in immature avian auditory hair cells, Ca_*V*_3.1 T-type subunits (Levic and Dulon, 2012).

## Discussion

In the present study, we compared the level of expression of the Ca^2+^ current in hair cells of the crista and cochlear IHCs from wild-type and *Ca_*V*_1.3^–/–^* mice. We also investigated whether the normal developmental acquisition of the mature-like K^+^ current in the crista hair cells was dependent on the presence of Ca_*V*_1.3 Ca^2+^ channels, as previous demonstrated for the IHCs (Brandt et al., 2003; Jeng et al., 2020a). We found that the adult-like K^+^ currents in type I and type II hair cells were similarly present in both wild-type and *Ca_*V*_1.3^–/–^* mice. We also showed that the IHCs and crista hair cells from *Ca_*V*_1.3^–/–^* mice exhibit a residual Ca^2+^ current that was only < 20% to that of control cells (ranging between 12 and 19% depending on hair-cell type and age investigated). The data provided have highlighted some differences in the way inner ear organs regulate the development of hair cells.

It is well established that the development of IHCs and outer hair cells (OHCs) in the cochlea is tightly linked to their ability to elicit Ca^2+^ dependent action potentials during pre-hearing stages (Corns et al., 2014; Ceriani et al., 2019), where hearing onset occurs at P12 in most altricial rodents. One of the basolateral biophysical characteristics of cochlear hair cells, which is controlled by this spiking activity, is the expression of adult-like K^+^ channels. Cochlear hair cells from pre-hearing *Ca_*V*_1.3^–/–^* mice, which are unable to elicit Ca^2+^ action potentials retain an immature K^+^ current profile even at adult stages (Brandt et al., 2003; Ceriani et al., 2019; Jeng et al., 2020a). We found that the absence of the Ca_*V*_1.3 Ca^2+^ current does not, however, influence the maturation of vestibular hair cells, most likely because these cells seem not to elicit action potentials during early postnatal developmental stages.

The absence of Ca_*V*_1.3 Ca^2+^ channels in both mice (Platzer et al., 2000; Dou et al., 2004) and humans (Baig et al., 2011) is associated with deafness but not visible vestibular dysfunctions. Thus, it is not clear why vestibular function is not suppressed or largely reduced by the absence of Ca^2+^-dependent exocytosis in hair cells. Different from hearing, partial vestibular compensation may occur by other sensory systems (e.g., proprioception and vision), so that specific vestibular tests such as vestibular evoked potentials (VsEPs) are likely to be required to unveil subtle vestibular impairments (Jones and Jones, 2014). This has been previously demonstrated for another synaptic protein, otoferlin, which is expressed in both cochlear and vestibular hair cells. Otoferlin knockout mice are deaf but do not show gross vestibular dysfunction (abnormal posturing, imbalance or nystagmus; Roux et al., 2006), although subtle vestibular deficits can be unveiled by VsEPs (Dulon et al., 2009).

Similar to cochlear IHCs, type I and type II hair cells from the crista of *Ca_*V*_1.3^–/–^* mice exhibit a small (<20%) residual Ca^2+^ current, indicating that the Ca_*V*_1.3 subunit carries the large majority of the current. This result differs from a previous study performed on early postnatal hair cells from the utricle of *Ca_*V*_1.3^–/–^* mice (P1–P10: Dou et al., 2004), which exhibit a much larger (about 50%) residual inward Ba^2+^ current through voltage-gated Ca^2+^ channels. It is unlikely that age difference among studies is the reason for this discrepancy because we observed a similar residual Ca^2+^ current in both neonatal and adult vestibular hair cells from *Ca_*V*_1.3^–/–^* mice. An alternative explanation is that the expression level of the Ca_*V*_1.3 subunit is different between utricle and crista hair cells. In principle, the residual Ca^2+^ current in some type I and type II vestibular hair cells could at least in part reduce the vestibular phenotype in *Ca_*V*_1.3^–/–^* mice, provided that the remaining Ca^2+^ channels are capable of driving some exocytosis. An attractive possibility for the absence of vestibular phenotypes in *Ca_*V*_1.3^–/–^* mice, however, is likely to be associated with the presence of non-quantal synaptic transmission in vestibular type I hair cells, but not cochlear IHCs (Yamashita and Ohmori, 1990; Holt et al., 2007; Songer and Eatock, 2013). This non-conventional, K^+^-based synaptic transmission relays on intercellular K^+^ increase in the extended narrow synaptic cleft between the basolateral membrane of type I hair cells and the inner face of the afferent calyx terminal (Lim et al., 2011; Contini et al., 2012, 2017; Spaiardi et al., 2020a) and fast, direct resistive coupling between pre- and postsynaptic K^+^ channels facing the synaptic cleft (Contini et al., 2020). *I*_*K,L*_, which is normally present in *Ca_*V*_1.3^–/–^* mice, plays a major role in this K^+^-based signal transmission at the calyx synapse (Eatock, 2018). Indeed, we found that intercellular K^+^ increase still occurs in *Ca_*V*_1.3^–/–^* mice (Figure 1), suggesting that calyces develop normally around the type I hair cell basolateral membrane. Non-synaptic activation of the calyx has also been described during the mechanical deflection of the hair bundle of the type I hair cells (Songer and Eatock, 2013). The presence of this K^+^-based afferent transmission, which is unlikely to be affected in *Ca_*V*_1.3^–/–^* mice, might contribute to the absence of obvious vestibular dysfunction in these mice.

## Data Availability Statement

The original contributions presented in the study are included in the article/supplementary material, further inquiries can be directed to the corresponding author/s.

## Ethics Statement

The animal study was reviewed and approved by University of Sheffield Ethical Review Committee.

## Author Contributions

WM and SM conceived and coordinated the study. All authors helped with the collection and analysis of the data.

## Conflict of Interest

The authors declare that the research was conducted in the absence of any commercial or financial relationships that could be construed as a potential conflict of interest.

## Publisher’s Note

All claims expressed in this article are solely those of the authors and do not necessarily represent those of their affiliated organizations, or those of the publisher, the editors and the reviewers. Any product that may be evaluated in this article, or claim that may be made by its manufacturer, is not guaranteed or endorsed by the publisher.
